# 13th Annual Biotechnology Congress (BAC Madrid 2019): abstract collection: Madrid, Spain. 10-12 July 2019

**DOI:** 10.1186/s12896-019-0569-8

**Published:** 2019-12-04

Introduction:

Aitor Balmaseda and Pablo Revilla

Spanish Federation of Biotechnologist, Campus of Vegazana, s / n, 24071 León

From 10th to 12th of July of this year the Spanish Federation of Biotechnologists (https://febiotec.es/) organized the 13th edition of the Annual Biotechnology Congress (BAC).

This event, held in Madrid, allowed young students and professionals to share their results and knowledge with other students and senior professionals, part of the audience, being the setting of this network the purpose of BAC Madrid 2019. Biotechnologists from all over Spain could meet to see the last trends thank to lectures from academia researchers, but also business biotechnology. Participants could also share with the audience their results through oral communications and posters.

A sample of abstracts of those communications presented in BAC Madrid may be found bellow. We are looking forward to seeing you in the next edition of our Congress which will take place in Zaragoza in July 2020.

## O1. Improvement of stability and activity under drastic conditions through different immobilization protocols for Polygalacturonase from *A. niger*

### Lucas Dal Magro^1,2^, Jakub F. Kornecki^1,4^, Manuela P. Klein^3^, Rafael C. Rodrigues^2^, Roberto Fernandez-Lafuente^1^

#### ^1^Department of Biocatalysis, ICP-CSIC, Campus UAM-CSIC, Cantoblanco, ZC 28049, Madrid, Spain; ^2^Biotechnology, Bioprocess and Biocatalysis Group, Institute of Food Science and Technology, Federal University of Rio Grande do Sul, Av. Bento Gonçalves, 9500, P.O. Box 15090, ZC 91501-970, Porto Alegre, RS, Brazil; ^3^Department of Nutrition, Federal University of Health Sciences of Porto Alegre (UFCSPA), ZC 90050-170, Porto Alegre, RS, Brazil; ^4^Orion High Technologies Calle Turín 15, Nave 3, 28983 Parla, Madrid

##### **Correspondence:** Jakub F. Kornecki (yakokornecki@hotmail.es)

Polygalacturonases (PGs) are enzymes that act over the pectic acid eliminating the α-1,4 bond between two non-esterified galacturonic acid residues. PGs from fungi present a high rate of activity and they perform best at slightly acidic pH values and an optimum temperature between 30°C and 50°C. The addition of PGs during juices production is focused on the degradation of pectin and other big molecules, reducing its viscosity and achieving a clear aspect for the juice, besides increasing the juice production and halving the required filtration time. PG from *Aspergillus niger* was immobilized using three different supports: glyoxyl, vinylsulfone and glutaraldehyde-activated amino support. The use of supports pre-activated with glutaraldehyde had the best results. PG immobilization was carried for 24h at pH 5, and at pH 5, 6.5 and 8 for 3h, and passed this time they were switched to pH 8 to complete the 24h. Another protocol used pH 8 adding 300 mM NaCl to prevent ionic exchange between the enzyme and the support. The immobilization under all conditions produced a significant increase in thermal stability during stress inactivation experiments at pHs from 4, up to 10. This permitted that at temperatures over 70°C or pH values that went over 7, the biocatalyst maintained significant levels of activity while the free enzyme was completely inactive. The immobilization conditions were key over enzyme activity, thermostability and operational stability, making us think that the different conditions applied, allowed PG to have different orientations while being immobilized. The interest on the performance of each biocatalyst depends on the parameter of most value (activity or stability) and the conditions used during the reaction. Optimal PG immobilized biocatalysts could be reused up to ten times without significant losses in enzyme activity and offered a very linear reaction courses.

Funding: This work was supported by grants and scholarships (L. Dal Magro) from Capes, CNPq (process 403505/2013-5) and FAPERGS (process 17/2551-0000939-8). We also gratefully recognize the economic support from the Comunidad Autónoma de Madrid (project Ref. IND2017/IND-7640) and the MICIU from Spanish Government, (project number CTQ2017-86170-R). The authors wish to thank Amazon group and LNF Latinoamericana for kindly supplying the enzymes used in this research.

## O2. Stable HEK293 cell line generation by CRISPR/Cas9 for the production of GagGFP VLPs

### Laia Bosch-Molist, Arnau Boix-Besora, Laura Cervera-Gràcia, Francesc Gòdia-Casablancas

#### Universitat Autònoma de Barcelona (UAB)

##### **Correspondence:** Laia Bosch-Molist (laiaboschm@gmail.com)

Virus-like particles (VLPs) are nanostructures that mimic the natural configuration of a virus [1]. They are based on the intrinsic ability of structural viral proteins to self-assemble into particles. Their capacity of generating a strong cellular and humoral immune response due to their repetitive subunits and not containing viral genetic material makes them good vaccine candidates [2]. HIV-1 VLPs are based on the polyprotein Gag which can form spherical structures when recombinantly expressed.

In this work, mammalian cell platforms are the selected systems for such complex and enveloped VLPs. This approach allows the incorporation of accurate post translational modifications into the VLP, which are important for vaccine efficacy. Production of recombinant Gag VLPs in HEK293 cultures can be achieved by transient gene expression (TGE) or stable gene expression (SGE) [3]. In TGE expression of the gene of interest is lost over time due to dilution in each cell division while SGE achieves a constitutive gene expression via direct integration of the gene of interest into the genome. CRISPR/Cas9 system introduces targeted double-stranded breaks (DSB) which may be repaired by homology-directed-repair (HDR) if a DNA template is used [4]. In here, we present an approach where HDR-mediated knock-in is used to generate an HIV-1 GagEGFP HEK293 stable cell line into the genomic safe harbour AAVS1.

References

[1] N. Kushnir, S. J. Streatfield, and V. Yusibov, “Virus-like particles as a highly efficient vaccine platform: Diversity of targets and production systems and advances in clinical development,” *Vaccine*, vol. 31, no. 1, pp. 58–83, 2012.

[2] W. A. Rodríguez-Limas, K. Sekar, and K. E. J. Tyo, “Virus-like particles: The future of microbial factories and cell-free systems as platforms for vaccine development,” *Curr. Opin. Biotechnol.*, vol. 24, no. 6, pp. 1089–1093, 2013.

[3] L. Cervera, S. Gutiérrez-Granados, N. S. Berrow, M. M. Segura, and F. Gòdia, “Extended gene expression by medium exchange and repeated transient transfection for recombinant protein production enhancement,” *Biotechnol. Bioeng.*, vol. 112, no. 5, pp. 934–946, 2015.

[4] D. Paquet *et al.*, “Efficient introduction of specific homozygous and heterozygous mutations using CRISPR/Cas9,” *Nature*, vol. 533, no. 7601, 2016.

## O3. Virtual Biopsy: A non-invasive technique for a specifically tumour detection

### González-Gómez, R, Ruiz-López, E., Torres-Herrero, B., Schuhmacher, AJ

#### Aragon Health Research Institute (IIS Aragón)

##### **Correspondence:** González-Gómez, R. (ruthgo1994@gmail.com)

Despite the survival rate of cancer patients is higher than the last decades, new diagnosis tools are needed for the appropriate detection of tumours. ImmunoPET is a visual technique where antibodies are used to specifically detect a protein which is expressed in tumours, what allows to make an efficient diagnosis in a non-invasive manner [1].

The best candidate for this technique is a protein that is differentially expressed between normal and tumoral tissue. By bioinformatic analysis, ABCC3 showed to be a good target for immunoPET in Glioblastoma Multiforme (GBM), patient data revealed poor survival with high levels of ABCC3.

ABCC3 is a member of the subfamily of MRP (multi-drug resistance proteins) that belongs to ATP-binding cassettes (ABC) transporters, it may play a role in organic anions excretion in liver and intestine [2].

We aim to determine a correlation between ABCC3 expression and chemotherapy response. Our studies began with a cell line with high ABCC3 expression levels, we generate ABCC3 knock-out cells and they were more sensitive to cis-platin and temozolomide treatment compared with wild type cells. GBM cell lines were characterized at mRNA and protein levels of ABCC3, showing different expressing profiles.

ABCC3 overexpressing and knock-out cells of GBM cell lines were developed, demonstrating that the lack of ABCC3 *in vitro* causes sensitivity of the cells to chemotherapeutics, likewise high protein levels confer resistance to drugs.

O6-Benzylguanine (O6-BG) is a potent inhibitor of O6-methylguanine-DNA methyltransferase (MGMT). When inhibiting MGMT, cells are more sensitive to temozolomide administration [3]. Combination treatment between cis-platin or temozolomide with O6-BG did not synergize, however MGMT levels were altered when ABCC3 is knocked-out.

It would be interesting to further investigate ABCC3 and MGMT relation for treatment improvement. ABCC3 could be a favourable target for immunoPET to improve diagnosis and determine chemotherapy response in different tissues.

References

1. Bailly C, Cléry PF, Faivre-Chauvet A, Bourgeois M, Guérard F, Haddad F, Barbet J, Chérel M, Kraeber-Bodéré F, Carlier T, Bodet-Milin C. Immuno-PET for Clinical Theranostic Approaches. *Internal Journal of Molecular Sciences*. 2016 Dec 28;18(1):57.

2. Vasiliou, V., Vasiliou, K. and Nebert, DW. (2009). Human ATP-binding cassette (ABC) transporter family. *Human Genomics* 3(3): 281-290

3. Cai, W., Maldonado, NV., Cui, W., Harutyunyan, N., Ji, L., Sposto, R., Reynolds, CP. And Keshelava, N. (2010). Activity of irinotecan and temozolomide in the presence of O^6^-methylguanine-DNA methyltransferase inhibition in neuroblastoma pre-clinical models. *British Journal of Cancer* 103(9): 1369-1379.

Funding: FERO Foundation, ASPANOA, Carlos III Health Institute, Ramón y Cajal program.

## O4. Crossing the blood-brain barrier: development of molecular imaging nanoprobes for non-invasive diagnosis of glioblastoma

### Eduardo Ruiz-López, Ruth González-Gómez, Beatriz Torres-Herrero, Alberto J Schuhmacher

#### Aragon Health Research Institute (IIS ARAGON), Molecular Oncology Group, Zaragoza, Spain

##### **Correspondence:** Eduardo Ruiz-López (educalata21@gmail.com)

Glioblastoma (GBM) is the most prevalent and most aggressive brain tumor. Current techniques for GBM diagnosis -Magnetic Resonance Imaging- still being unaccurate and molecular imaging approaches are emmerging as non-invasive tools. Targeting of GBM with highly specific imaging probes allows non-invasive diagnosis, treatment stratification and monitoring of patients. Overexpressed GBM biomarkers have allowed the desing of monoclonal antibodies for molecular imaging. However, their large size impedes the penetration through the blood-brain barrier (BBB) and diffusion into the tumor. Smaller protein scaffold-based probes targeting GBM are being developed [*target*]: nanobodies [*HGF*], affibodies [*PDGFRβ, EGFR, IGF-1R, VEGFR2*], knottins [*phosphatidyl inositide, MMP-2, integrins αvβ3, αvβ5*], anticalins [*HGF, VEGF-A*], adnectins [*VEGFR*] and DARPins [*HGF*]. Their lower molecular weight enables better tumor uptake and faster clearance compared with monoclonal antibodies. Successful molecular imaging has been conducted on preclinical xenograft and orthotopic mice models.

We have bioinformatically identified molecular targets for the development of molecular imaging probes for GBM. We are developing nanobody-based targeting agents against these candidates following two strategies. First, we are actively immunizing dromedaries with a target-overexpression glioma cell line in order to clone the targeted-enriched repertoire of variable domain of camelid heavy chain. We will generate a nanobody-expressed bacteria library or nanobody *nanothèque*. Bacteria expressing high affinity nanobodies will be selected by *E. coli* display. Also, we are isolating nanobodies against GBM target from a gene library by phage display. Selected nanobody probes will be used for molecular imaging of xenograft and “avatar” mice models. For clinical development, we will fusion our nanobodies with molecular BBB shuttles (ApoE, FC5 nanobody) to improve brain access via transcytosis. Multitargeting nanobodies, dual labeling strategies and radionuclide theragnostics approaches are considered. In the future, genetic profile of patients may allow molecular personalized immunotargeting and same-day imaging of GBM.

Funding: Asociación Española contra el Cáncer (AECC), Programa Ramón y Cajal (RyC) y Fundación de Investigación Oncológica (FERO).

## P1. Antibacterial activity of herbal extracts against Gram-positive and Gram-negative bacteria

### Carla Michelle Parada Sosa^1,2^, Rosa del Carmen Rocha Gracia^3^, Magdalena Martínez Cañamero^4^, Antonio Cobo Molinos^5^ and Zaida Nelly Juárez^1^

#### ^1^Facultad de Biotecnología. Decanato de Ciencias Biológicas, Universidad Popular Autónoma del Estado de Puebla, 21 Sur 1103, Colonia Santiago, 72410 Puebla, Puebla, México; ^2^Facultad de Ciencias Experimentales, Universidad de Jaén, 23071-Jaén, España; ^3^Centro de Investigaciones en Ciencias Microbiológicas, ICUAP, Benemérita Universidad Autónoma de Puebla. Complejo de Ciencias, edificio No. 103 J. Ciudad Universitaria, Colonia San Manuel, 72570 Puebla, México; ^4^Área de Microbiología, Departamento de Ciencias de la Salud, Facultad de Ciencias Experimentales, Universidad de Jaén, 23071-Jaén, España; ^5^Departamento de Microbiología, Facultad de Ciencias, Universidad de Granada, 18071-Granada, España

##### **Correspondence:** Carla Michelle Parada Sosa (carlamichelle.parada@upaep.edu.mx)

Antimicrobial resistance (AMR) spread is an alarming global health issue. Bacterial infections are not only linked to the hospital area, they are also an issue in the food area. A strategy against AMR is to strengthen the research on alternative antimicrobials [1].

Herbal extracts and their secondary metabolites are an option for treating infections or preserving food [2].

In this study, evaluations of antimicrobial activities of herbal extracts were carried out using *in vitro* antimicrobial susceptibility assays against Gram-positive and Gram-negative strains in order to propose the active samples as antimicrobials. Extracts of *A. ludoviciana, Helianthus annuus* var*. Autumn Beauty* y *Lycopodium clavatum* showed antibacterial properties against Gram-positive and Gram-negative strains. This property of herbal extracts could be an alternative for finding natural antimicrobials for future research and development of new products to treat hospital infections, food poisoning or as food preservatives.

References

1. Blair JMA, Webber MA, Baylay AJ, Ogbolu DO, Piddock LJ V. Molecular mechanisms of antibiotic resistance. Nat Rev Microbiol. 2015;13(1):42–51.

2. Yang S, Low L, Yap PS, Yusoff K, Mai C, Lai K, et al. Plant-Derived Antimicrobials : Insights into Mitigation of Antimicrobial Resistance. Rec Nat Prod. 2018;4:1–22.

## P2. Evaluation of protective effect of agroalimentary industry by-products on a mucosal barrier model

### Javier Quero^1^, Inés Mármol^1^, Raquel Ibarz^2^, Olga Martín-Belloso^2^ and Mª Jesús Rodríguez-Yoldi^1^

#### ^1^ Pharmacology and Physiology Department, Faculty of Veterinary, University of Zaragoza, Zaragoza, Spain; ^2^ Food Technology Department, University of Lleida, Agrotecnio Center, Spain

##### **Correspondence:** Javier Quero (javierquero94@gmail.com)

Oxidative stress on intestinal mucosal barrier is closely related to the development of a wide range of chronic disorders such as Inflamatory Bowel Diseases and colorectal cancer (CRC). This exogenous oxidative stress can be triggered by multiple external factors such as diet, drugs, smoking or alcoholism, which increase reactive oxygen species (ROS) and reactive nitrogen species (RNS) in the intestinal tissue [1,2].

Fruits and vegetables are well-known for their antioxidant potential given their composition in phenolic compounds and other bioactive compounds [3], which can be also obtained from forest and agro-industrial by-products.

We have analyzed the potential use of agroalimentary industry waste as protective agents for the mucosal barrier upon oxidative stress induction. In addition, influence of maltodextrin-encapsulation has been evaluated. The selected cellular intestinal barrier model were differentiated Caco-2 cells over 80% confluence, which were exposed to 10 mM H_2_O_2_ for 20 minutes.

Results suggest the potential role of agro-food by-products as protective agents for the intestinal barrier upon exogenous oxidative stress induction. Therefore, food waste might be of interest for the pharmaceutical industry on the management of oxidative stress-related diseases of the intestinal barrier, giving a second life to a great amount of waste generated by forest and agro-food industries.

References

[1] Reuter, S. *et al.* Oxidative stress, inflammation, and cancer: How are they linked? *Free Radic Biol Med* (2010) 49(11), 1603-1616.

[2] Perse, M. Oxidative stress in the pathogenesis of colorectal cancer: cause or consequence? *Biomed Res Int* (2013) 2013, 725710-725719.

[3] Tsao, R. Chemistry and biochemistry of dietary polyphenols *Nutrients* (2010) 2, 1231-1246.

Funding: Ministerio de Ciencia e Innovación (SAF2016-75441-R), Gobierno de Aragón/European Regional Development Fund (ERDF) (B16-R17) and Interreg Sudoe REDVALUE (SOE1/PI/E0123)

## P3. Evaluation of *p*-benzoquinone and divinylsulfone as support activating agents for lipase stabilization

### Nathalia Saraiva Rios^1,4^, Davino M. Andrade Neto^2^, José Cleiton Sousa dos Santos^3^,Pierre Basílio Almeida Fechine^2^, Roberto Fernández-Lafuente^4^, Luciana Rocha Barros Gonçalves^1^

#### ^1^Departamento de Engenharia Química, Universidade Federal do Ceará, Campus do Pici, Bloco 709, CEP 60455-760, Fortaleza, CE, Brazil; ^2^Departamento de Química Analítica e Físico-Química, Centro de Ciências, Universidade Federal do Ceará, Av. Mister Hull s/n, Pici, 60455-760, Fortaleza,CECP12200, Brazil; ^3^Instituto de Engenharias e Desenvolvimento Sustentável, Universidade da Integração Internacional da Lusofonia Afro-Brasileira, 62785000 Acarape, CE, Brazil; ^4^Departamento de Biocatálisis. ICP-CSIC, Campus UAM-CSIC, Madrid, Spain

##### **Correspondence:** Nathalia Saraiva Rios (nathaliarios25@yahoo.com.br)

Lipases have been widely used in food industry, chemical and pharmaceutical processes, because their high activity and high chemo-, regio- and enantio-selectivity [1]. Therefore, the immobilization of enzymes has commonly used, since it enables the easy separation of the reaction medium and the reuse of the biocatalyst [2]. In this context, a novel NiZnFe_2_O_4_ superparamagnetic nanoparticle was used as support for immobilization of the lipase from *Pseudomonas fluorescens* (PFL). For this purpose, this nanoparticle was coated with silica by impregnation with tetraethoxysilane (TEOS) and further activated with *p*-benzoquinone (BQ) and divinylsulfone (DVS), to produce the covalent immobilized biocatalysts. BQ and DVS are bifunctional molecules that can react with different moieties of enzyme (amine, hydroxyl, thiol and among others), producing highly stable biocatalysts [3,4]. For the best of our knowledge, BQ has not been properly studied to stabilize enzymes via multipoint covalent attachment. Therefore, we use this activating agent to immobilize PFL comparing with DVS, which is a promising support activator. PFL also immobilized on TEOS coated NiZnFe_2_O_4_ nanoparticles without any activation to produce the adsorbed immobilized biocatalyst. BQ biocatalyst demonstrated the most active and stable preparation (Recovered activity: 89 % and t_1/2_(60°C) over 1440 min), while the DVS covalent and adsorbed preparation presented a recovered activity of 82 and 55 % and half-lives of 225 and 150 min at 60°C, respectively. Therefore, this study shows a successful immobilization strategy, producing active and stable biocatalysts.

References

[1] D. Sharma, B. Sharma, A. K. Shukla, Biotechnological approach of microbial lipase: A review, Biotechnology. 10 (2011) 23–40.

[2] R. A. Sheldon, S. van Pelt, Enzyme immobilisation in biocatalysis: why, what and how., Chem. Soc. Rev. 42 (2013) 6223–6235.

[3] J.C.S. dos Santos, N. Rueda, O. Barbosa, J.F. Fernández-Sánchez, A.L. Medina-Castillo, T. Ramón-Márquez, M.C. Arias-Martos, M.C. Millán-Linares, J. Pedroche, M. del M. Yust, L.R.B. Gonçalves, R. Fernandez-Lafuente, Characterization of supports activated with divinyl sulfone as a tool to immobilize and stabilize enzymes via multipoint covalent attachment. Application to chymotrypsin, RSC Adv. 5 (2015) 20639–20649.

[4] N.S. Rios, D.M. Andrade Neto, J.C.S. dos Santos, P.B.A. Fechine, R. Fernández-lafuente, L.R.B. Gonçalves, Comparison of the immobilization of lipase from Pseudomonas fluorescens on divinylsulfone or p -benzoquinone activated support, Int. J. Biol. Macromol. 134 (2019) 936–945.

Funding: Nathalia S. Rios thanks to CNPq for a predoctoral fellowship (CNPq scholarship – Brazil). MICIU from Spanish Government, (project number CTQ2017-86170-R).

## P4. *Clostridium tyrobutyricum* detection by real time PCR and specific high affinity ligands

### Miriam Esteban^1^, Juan Pedro Navarro^1^, Patricia Galán-Malo^2^, Luis Mata^2^, Mª Dolores Pérez^1^, Lourdes Sánchez^1^

#### ^1^ Departamento de Producción Animal y Ciencia de los Alimentos. Facultad de Veterinaria. Instituto Agroalimentario de Aragón (IA2) (Universidad de Zaragoza-CITA), Zaragoza, Spain; ^2^ ZEULAB S.L., Zaragoza, Spain

##### **Correspondence:** Miriam Esteban (miriames93@gmail.com)

*Clostridium tyrobutyricum* is the major agent that causes the blowing defect problem in cheeses due to the germination of its dormant spores during the ripening [1]. As a result of the pressure exerted by the gases produced, many of the affected cheeses show cavities and cracks, which cause the product loss in most cases.

Our aim is to develop a fast method to detect the spores in milk instead of the traditional microbiological ones that are not specific and are time consuming. For this reason, we developed an approach based on previous digestion of milk with subtilisin for recovering the spores. For the real time PCR, we tested different methods for disruption of spores, which is a total challenge due to its resistant structure. The microwave treatment followed by a standard DNA purification has been found to be the most efficient disruption procedure. We were able to detect from 10^1^ spores/mL to 10^7^ spores/mL, but we were not able to distinguish the quantity because of the low DNA recovery and also due to the effect of SASPs proteins on DNA packaging [2].

On the other hand, we *C. tyrobutyricum* spores detection by flow cytometry because it has the advantage of not disrupting the spores. The detection was done with a high affinity ligand as an alternative to antibodies produced in animals. The ligand was developed previously by Phage Display and its affinity for butyric spores analyzed by isothermal titration calorimetry (ITC). These results showed good binding of *C. tyrobutyricum* spores, but also for other clostridium species. Flow cytometry with specific ligands showed good results with a limit of detection of 10^3^ spores/mL and could be an alternative to the real time PCR to avoid the problem of DNA recovery from spores.

References

[1] Bassi, D., Puglisi, E., Cocconcelli, P.S., 2015. Understanding the bacterial communities of hard cheese with blowing defect. Food Microbiol. 52, 106–118. https://doi.org/10.1016/j.fm.2015.07.004

[2] Raju, D., Waters, M., Setlow, P., Sarker, M.R., 2006. Investigating the role of small, acid-soluble spore proteins (SASPs) in the resistance of Clostridium perfringens spores to heat. BMC Microbiol. 13.

Funding: Aragon Government (Spain) and the European Social Fund under a DGA predoctoral grant and by the AGL2013-44130-R project financed by the Ministerio de Ciencia e Innovación of the Spanish Government.

## P5. Biotechnological potential and prospective for sustainability of bacteria isolated from soil and rhizosphere of cover crops in an olive grove

### Marta Pérez-Díaz^1^, Elena G. Biosca^2^, Belén Álvarez^1^

#### ^1^ IMIDRA, A2 km 38.200, 28800 Alcalá de Henares, Madrid, Spain; ^2^ University of Valencia, Avda. Dr. Moliner 50, 46100 Burjasot, Valencia, Spain

##### **Correspondence:** Belén Álvarez (mariabelen.alvarez@madrid.org)

Large-scale production of environmental microorganisms to exploit their biotechnological applications can be of great interest in agri-food industry. Activities of microorganisms with biotechnological use are the production of hydrolytic exoenzymes with diverse functions, the ability to supply essential nutrients to plants acting as biofertilizers, and the antagonistic effects they can exert against pathogenic microorganisms [1,2]. The beneficial consequences of their application can contribute to achieve a microbe-based sustainable agriculture [3].

A collection of soil and rhizosphere bacteria was isolated from cover crops of an olive grove in mainland Spain. All of them were identified and tested for their potential to produce exoenzymes related to the degradation and recycling of organic compounds, their ability to solubilize phosphates, fix atmospheric nitrogen, and capture iron, and their antagonism against relevant phytopathogenic fungi such as *Verticillium dahliae*, *Phaeomoniella chlamydospora*, *Dactylonectria macrodidyma* and *Fusarium avenaceum*.

Molecular identification of the isolates revealed a majority of *Bacillus* spp., and also *Massilia* spp., *Brevibacterium* spp., *Pantoea* spp., *Pseudomonas* spp., *Enterococcus* spp. and *Chryseobacterium* spp. as the main genera present. Production of exoenzymes as proteases, lipases, amylases, and DNases was positive for 96% of the isolates, whereas 92% was positive for phosphate solubilization, nitrogen fixation and/or iron uptake, and 41% displayed effective antagonism against, at least, one of the fungal pathogens tested. *V. dahliae* growth was inhibited by 33% of the isolates, one of them also by production of volatile organic compounds (VOCs). Inhibitory activity was detected against *P. chlamydospora* in 18% of the isolates and *D. macrodidyma* in 4%, without production of VOCs but, not against *F. avenaceum*. The high activity observed in soil and rhizosphere bacteria of the cover crops in the olive grove suggests a great potential to be produced in the biotechnological industry [4] for exoenzyme production, recycling of nutrients, reduction of agrochemicals, and/or biological control.

References

^1^ Abada E, Al-Faifi Z, Osman M. 2017. Enzymes and nanoparticles produced by microorganisms and their applications in biotechnology. In: Fungal nanotechnology, fungal biology. Prasad R (Ed). Springer International Publishing. Doi: 10.1007/978-3-319-68424-6_7.

^2^ Parnell JJ, Berka R, Young HA, Sturino JM, Kang Y, Barnhart DM, DiLeo MV. 2016. From the lab to the farm: an industrial perspective of plant beneficial microorganisms. Front. Plant Sci. 7:1110. Doi: 10.3389/fpls.2016.01110.

^3^ Umesha S, Singh PK, Singh RP. 2018. Microbial biotechnology and sustainable agriculture. In: Biotechnology for sustainable agriculture. Emerging approaches and strategies. pp. 185-205. Elsevier.

^4^ Prasad R, Gill SS, Tuteja N (Eds). 2018. New and future developments in microbial biotechnology and bioengineering. Crop improvement through microbial biotechnology. Elsevier. Doi: 10.1016/C2016-0-04330-9.

Funding: This work was funded by the AGRISOST S2013/ABI-2717 project, and BACPLANT-UVEG-Research Support

## P6. Modifying the immobilization conditions of TLLs on octyl agarose beads to modulate their catalytic properties

### Sara Arana-Peña^1^, Yuliya Lokha^1^, Nathalia S. Rios^1,2^, Carmen Méndez- Sánchez^1^ and Roberto Fernández-Lafuente^1^

#### ^1^Departamento de Biocatálisis, Instituto de Catálisis y Petroleoquímica (ICP-CSIC), Campus UAM-CSIC, 28049 Madrid, Spain; ^2^Departamento de Engenharia Química, Universidade Federal do Ceará, Campus do Pici, CEP 60455-760, Fortaleza, CE, Brazil

##### **Correspondence:** Sara Arana-Peña (s.arana@csic.es)

Lipases are among the most used enzymes due to their unique properties and may be found in all living beings. Lipase immobilization is mainly necessary if biocatalyst’s recovery and reuse is desired, as well as improve its properties. The lipases immobilization at low ionic strength on hydrophobic supports allows single step immobilization via interfacial activation, locking the lipase open form, and giving it a high stabilization [1]. It may be expected that immobilization conditions can have a significant impact on lipase conformation. In fact, the lipase from *Thermomyces lanuginosus* (TLL) has been described as sensible to the changes of the immobilization conditions [2]. Thus, in this work we intend to investigate if reversible immobilization of this enzyme on hydrophobic support under different conditions, such as pH and presence of some ions, may have an effect on their final stability and catalytic activity. Phosphate ions have proved to decrease the stability of many lipases [3], on the other hand, some cations (e.g., Ca2+) may increase enzyme stability, but mostly when immobilized on hydrophobic supports [4]. Octyl-agarose beads have been chosen as support, as they are widely used and agarose has some good properties. After performing the assays, we confirmed that immobilization conditions, using one lipase and the same support, can alter significantly the final catalytic activity and stability of TLL.

References

[1] J.M. Palomo, G. Muñoz, G. Fernández-Lorente, C. Mateo, R. Fernández-Lafuente, J. Guisan, Interfacial adsorption of lipases on very hydrophobic support (octadecyl-Sepabeads): Immobilization, hyperactivation and stabilization of the open form of lipases, J. Mol. Catal. B Enzym. 19 (2002) 279–286. doi:10.1016/S1381-1177(02)00178-9.

[2] E. Abreu Silveira, S. Moreno-Perez, A. Basso, S. Serban, R. Pestana Mamede, P.W. Tardioli, C. Sanchez Farinas, J. Rocha-Martin, G. Fernandez-Lorente, J.M. Guisan, Modulation of the regioselectivity of Thermomyces lanuginosus lipase via biocatalyst engineering for the Ethanolysis of oil in fully anhydrous medium, BMC Biotechnol. 17 (2017) 88. doi:10.1186/s12896-017-0407-9.

[3] S. Arana-Peña, Y. Lokha, R. Fernández-Lafuente, Immobilization of Eversa Lipase on Octyl Agarose Beads and Preliminary Characterization of Stability and Activity Features, Catalysts. 8 (2018) 511. doi:10.3390/catal8110511.

[4] L. Fernandez-Lopez, R. Bartolome-Cabrero, M.D. Rodriguez, C.S. Dos Santos, N. Rueda, R. Fernandez-Lafuente, Stabilizing effects of cations on lipases depend on the immobilization protocol, RSC Adv. 5 (2015) 83868–83875. doi:10.1039/C5RA18344H.

Funding: MICIU from Spanish Government, (project number CTQ2017-86170-R)

## P7. Amination a tool to improve the stabilisation of ficin by immobilization on glyoxyl agarose

### El-Hocine Siar ^1,2^, Sara Arana-Peña^1^, Roberto Morellon-Sterling^1^. Jakub F. Kornecki^1^, Mohammed Nasreddine Zidoune^2^, Oveimar Barbosa^3^, Roberto Fernandez-Lafuente^1^

#### 1. Departamento de Biocatálisis, Instituto de Catálisis-CSIC, Campus UAM-CSIC, 28049 Madrid, Spain; 2. Equipe TEPA, Laboratoire LNTA, INATAA, Université des Frères Mentouri Constantine 1, 25000 Constantine, Algeria; 3. Departamento de Química, Facultad de Ciencias, Universidad del Tolima, 730006299 Ibagué, Colombia

##### **Correspondence:** El-Hocine Siar (hocines1@hotmail.fr)

Enzymes are very useful tools in the modern biotechnology, because of their specificity and selectivity in the green chemistry. But some properties limit their use (inhibition, specificity and possibility of reuse). Immobilization and chemical modification of the free or immobilized enzyme are among the most proposed solutions to overcome these limitations. In this work, we studied the effects of amination using carbodiimide and ethylenediamine on the properties of immobilized/stabilized ficin on glyoxyl agarose and free ficin. The free ficin was first aminated then immobilized on glyoxyl agarose. Thus, the immobilized ficin on glyoxyl agarose was directly aminated. Ficin activity versus casein and BANA (benzoyl-arginine-*p*-nitroanilide) was determined and stability at different pH (5, 7 and 9) was checked. The activity of the aminated immobimized ficin was altered (versus casein and BANA), and while, activity versus casein slightly increased and versus BANA decreased. The heist effect was reported at pH 9, where activity vesus casein increased over 10% and activity versus BANA decreased more than 5 folds. Thus greatly altering the enzyme specificity. The amination improved the enzyme stability at pH 5 while stability was impoverished at pH 9. On the other hand, free aminated ficin, retained around 80% of activity versus BANA and 90% versus casein. After optimization of the immobilization protocol, the new biocatalyst was compared to the obtained using the non-aminated enzyme. Activity versus BANA decreases, but it increased versus casein. The aminated ficin biocatalyst was more stable than the non-aminated ficin biocatalyst mainly at pH 7. Amination of immobilized or free enzyme may be considered interesting for preparing improved biocatalyst of ficin, mainly in proteolytic applications. This may be also considered (mostly amination) as a possibility for further improves of enzyme immobilization.

Funding: We gratefully recognize the support from the MICIU from Spanish Government, (project number CTQ2017-86170-R) Mr. El-Hocine Siar thank Algerian Ministry of higher education and scientific research for their fellowships (Programme National Exceptionnel, Algeria P.N.E.

## P8. Acetylated chitosan surfaces for the development of a highly sensitive ELISA

### Tania García-Maceira^1^, Ana B. Aragón Gómez^1^, Verónica Luna-Guerrero^1^, Gracia Montero-Peñalvo^1^, Sara Gómez-Melero^1^, Fé I. García-Maceira^1^, José A González-Reyes^2^, Elier Paz-Rojas^1^

#### ^1^ Canvax Biotech, Córdoba, 14014, Spain ^2^ Department of Cell Biology, Physiology and Immunology, University of Córdoba, 14014, Spain

##### **Correspondence:** Tania García-Maceira (t.garcia@canvaxbiotech.com)

The enzyme-linked immunosorbent assay (ELISA), is the most widely used and reliable clinical routine method for the detection of important protein markers in healthcare. Several methods have been developed to improve the ELISA sensitivity through immobilization of antibodies on the microtiter plates [1]. We have developed a highly sensitive ELISA strategy based on the preparation of acetylated chitosan surfaces in order to improve the antibodies orientation. For this purpose, we used capture antibodies fused to the chitin binding domain (ChBD) of chitinase A1 from *Bacillus circulans* WL-12 [2]. Chitin surfaces were obtained by mixing small quantities of chitosan and acetic anhydride in each well of a microtiter plate and allowing the mixture dry overnight [3]. Anti-c-myc 9E10 low affinity antibody fused to ChBD was cloned and expressed in CHO cells obtaining the anti-c-myc-ChBD antibody. We demonstrated that anti c-myc-ChBD binds specifically to the chitin surfaces in comparison with anti-c-myc 9E10 which did not bind to it. Chitin surface was used to develop a sandwich ELISA to detect the chimeric protein human c-myc-GST-IL8 cloned and expressed in *Escherichia coli*. The limit of detection and quantification of the high binding protein microtiter and chitin-treated plates were compared. The ELISA procedure developed on chitin surface were 6-fold more sensitive than the ELISA performed on standard surface with significant differences (p<0.0001). Acetylated chitosan surfaces allow the antibody orientation on the surface and may be an appropriated method to replace the standard surfaces given the stability in time and the low cost of its preparation.

References

1. Welch NG, Scoble JA, Muir BW, Pigram PJ. Orientation and characterization of immobilized antibodies for improved immunoassays, Biointerphases, 2017; 12(2): 02D301.

2. Hashimoto M, Ikegami T, Seino S, Ohuchi N, Fukada H, Sugiyama J, Shirakawa M, Watanabe T. Expression and characterization of the Chitin-Binding Domain of Chitinase A1 from *Bacillus circulans* WL-12. Journal of bacteriology. 2000; 182(11):3045-3054.

3. Bernard MP, Cao D, Myers RV, Moyle WR. Tight attachment of chitin-binding-domain-tagged proteins to surfaces coated with acetylated chitosan. Analytical Biochemistrry. 2004; 327:278-283

Funding: Reference: 76359 Project: 360229 Junta de Andalucía, España

## P9. Dynamic gammagraphy evidences glomerular filtration in renal organoids developed after metanephroi transplantation

### Ximo García-Dominguez^1^, Pablo Sopena-Novales^2^, César D Vera-Donoso^3^, Juan V. Catret^4^, José S Vicente^1^, Francisco Marco-Jiménez^1^

#### ^1^ Institute for Animal Science and Technology, Universidad Politécnica de Valencia, 46022- Valencia, Spain; ^2^ Department of Nuclear Medicine, Hospital Universitari i Politècnic La Fe, 46026- Valencia, Spain; ^3^ Department of Urology, Hospital Universitari i Politècnic La Fe, 46026- Valencia, Spain; ^4^ Oncovision, 46022 - Valencia, Spain

##### **Correspondence:** Ximo García-Dominguez (ximo.garciadominguez@gmail.com)

The idea of generating a functional kidney graft on demand would extend the option of kidney transplantation to more patients with end-stage kidney disease. Transplantation of embryonic kidneys (metanephroi) showed that if these intact primordia were transplanted into adult hosts, it can mature attracting a new vascular system from host, avoiding immune response and exhibiting functional properties [1]. Our purpose was to determine the excretory functionality of the developed metanephroi using an *in vivo* renal scintigraphy, as well as post-mortem examination and histological evaluation.

With this aim, using the rabbit model, 14 metanephroi were micro-dissected from 15-day-old foetuses and transplanted into the retroperitoneal fat of 3 non-immunosuppressed hosts, using a minimally invasive laparoscopic technique [2]. Three weeks after transplantation, cortical renal scintigraphy was performed using the Sentinella® 102 and 3mCi of technetium-99m DMSA 30 minutes before planar acquisition of the images. After that, recipients were euthanized and organoids were retrieved, evaluating morphologically and histologically. Host kidneys were used as control.

The renal scintigraphy analysis demonstrated the renal function in new kidneys developed from transplanted metanephroi. Renal activity was 4 times lower for new kidneys in comparison with host kidneys (10000 vs 41000 counts, respectively). Nevertheless, new kidneys were 21 times smaller than host kidneys (0.5±0.44 vs 11.2 ±0.18 g, respectively). Moreover, this renal activity was in concordance with histological results, as glomeruli developed from these new kidneys were mature and similar to those present in host kidneys (40±4 vs 41±2 cells/glomerulus, respectively). In addition, the hydronephrosis state of the new kidneys as consequence of the lack of connection between the new ureter and the host's bladder, prove the excretory function of the new kidneys. Therefore, this study could pave a way to an unlimited supply of kidney for transplantation that could put an end to the current long waiting lists.

Funding: This research was supported by ALCER TURIA (association for the fight against kidney diseases) and anonymous donation to a crowdfunding campaign (Precipita platform, FECYT).

## P10. Melatonin supplementation benefits sperm functionality during capacitation

### Andrea Núñez-González^1,2^, Estela Fernández-Alegre^1,2^, Indira Álvarez-Fernández^1^, Amer Salman^1^, Juan Carlos Domínguez^1,3^, Beatriz Martín-Fernández^2^, Felipe Martínez-Pastor^1,4^

#### ^1^Institute of Animal Health and Cattle Development (INDEGSAL), León, 24071, Spain; ^2^Bianor Biotech, León, 24071, Spain; ^3^Department of Medicine, Surgery and Veterinary Anatomy, Universidad de León, León, 24071, Spain, ^4^Department of Molecular Biology, Universidad de León, León, 24071, Spain

##### **Correspondence:** Andrea Núñez-González (annugo95@gmail.com)

Melatonin is a ubiquitous molecule that regulates numerous physiological functions and plays an important role in male reproduction, directly influencing testosterone levels and sperm quality [1]. The presence of sperm membrane melatonin receptors in several animal species supports the hypothesis of a direct action of this molecule on spermatozoa [2]. Sperm maturation, capacitation and survival may thus be affected by the presence of endogenous melatonin. This study aimed to assess the direct influence of melatonin on mitochondrial activity and reactive oxygen species (ROS) production of sperm from bull (*Bos taurus taurus*) and Iberian red deer *(Cervus elaphus hispanicus*). Sperm samples from five deer and five bulls, epididymal and ejaculated respectively, were treated with different concentrations of melatonin (1 μM, 10 nM, 100 pM, 1 pM) and then incubated (39 °C, 5% CO_2_) for 4 h, under non-capacitating conditions (Hepes-buffered TALP) and capacitating conditions (Hepes-buffered TALP supplemented with 2 U/mL heparin). Flow cytometry analysis showed a significant increase (p<0.05) in the ratio of viable sperm cells with active mitochondria after melatonin treatment under capacitating conditions, for all tested concentrations. This effect was not seen when using non-capacitating conditions, except for 10 pM melatonin treatment on deer sperm, which could be a slightly capacitating environment in this species. Melatonin treatment under non-capacitating conditions lowered (p<0.05) the ratio of sperm cells with high mitochondrial superoxide production for all tested concentrations in both species. A similar effect was observed in deer sperm under capacitating conditions. Our results suggest that even the lowest concentrations of melatonin promote sperm survival during capacitation, presumably by stimulating signaling pathways through interaction with sperm membrane melatonin receptors. ROS function as second messengers for sperm capacitation [3], so the modulation of its levels could affect this process. The effect of melatonin on sperm cells may nevertheless vary considerably between species.

References

1. Li C, Zhou X. Melatonin and male reproduction. Clin Chim Acta. 2015;446:175–80.

2. Talpur H, Chandio I, Brohi R, Worku T, Rehman Z, Bhattarai D, et al. Research progress on the role of melatonin and its receptors in animal reproduction: A comprehensive review. Reprod Domest Anim. 2018;53(4):831–49.

3. de Lamirande E, O’Flaherty C. Sperm activation: Role of reactive oxygen species and kinases. Biochim Biophys Acta - Proteins Proteomics. 2008;1784(1):106–15.

Funding: This work was supported by MINECO (AGL2013-43328P and AGL2016-81890-REDT).

## P11. Self-assembling and tumor cell-targeting of protein nanoparticles controlled by modular engineering

### Eric Voltà-Durán ^1,2,3^, Olivia Cano-Garrido ^1,4^, Naroa Serna ^1,2,3^, Hèctor López-Laguna ^1,2,3^, Laura Sánchez-García ^1,2,3^, Mireia Pesarrodona ^1,2,3 ¥^, Ramón Mangues ^3,5^, Antonio Villaverde ^1,2,3^, Esther Vázquez ^1,2,3^, Ugutz Unzueta ^3,5^

#### ^1^ Institut de Biotecnologia i de Biomedicina, Universitat Autònoma de Barcelona, Bellaterra, 08193 Barcelona, Spain; ^2^ Departament de Genètica i de Microbiologia, Universitat Autònoma de Barcelona, Bellaterra, 08193 Barcelona, Spain; ^3^ CIBER de Bioingeniería, Biomateriales y Nanomedicina (CIBER-BBN), C/ Monforte de Lemos 3-5, 28029 Madrid, Spain; ^4^ Nanoligent SL, Edifici EUREKA, Universitat Autònoma de Barcelona, Bellaterra, 08193 Barcelona, Spain; ^5^ Institut d'Investigacions Biomèdiques Sant Pau and Josep Carreras Research Institute, Hospital de la Santa Creu i Sant Pau, 08041 Barcelona, Spain; ^¥^ Present address: Institute for Research in Biomedicine (IRB Barcelona), The Barcelona Institute of Science and Technology, 08028 Barcelona, Spain

##### **Correspondence:** Eric Voltà-Durán (eric.voltaduran@gmail.com)

The use of proteins as drug delivery systems is gaining interest in nanomedicine, especially when dealing with targeted therapies which require specific interaction and penetration into target cells [1]. By genetic fusion, proteins with multiple biological activities can be synthetized as single-chain polypeptides. This way, just a unique step of recombinant production is needed to obtain the final modular protein, in which each domain plays a determinant and distinct role [2]. In this context, the combination of both cationic peptides and polyhistidines into a protein structure has been proven as a universal self-assembling protein platform [3]. The paradigmatic example of this is the modular protein T22-GFP-H6, in which T22 (a potent ligand of the tumoral marker CXCR4) and H6 promote the formation of protein-only nanostructures around 11 nm (above renal filtration cut-off) which selectively bind and internalize CXCR4+ tumor cells *in vitro* and *in vivo* [4]. In here, we study the relevance of T22 and H6 positioning inside the modular structure by performing a morphological and functional characterization of alternatives in which T22 and H6 are rearranged in different locations. Interestingly, we prove that T22 must be placed at the amino terminus to allow CXCR4^+^ cell binding and specific internalization, while placing H6 at the carboxy terminus is crucial for promoting self-assembling. Moreover, the ability to oligomerize as regular protein nanoparticles increases cell penetrability in a cooperative way. Taken together, these data are of relevant interest for the design of smart nanoscale protein carriers for targeted drug delivery, as CXCR4 is a significant homing marker of many pathologies, such as many types of cancers and HIV infection, among others.

References

[1] Casanova I, Unzueta U, Arroyo-Solera I, Cespedes MV, Villaverde A, Mangues R, et al. Protein-driven nanomedicines in oncotherapy. Current opinion in pharmacology 2019;47:1-7.

[2] Sanchez-Garcia L, Martin L, Mangues R, Ferrer-Miralles N, Vazquez E, Villaverde A. Recombinant pharmaceuticals from microbial cells: a 2015 update. Microbial cell factories 2016;15:33.

[3] Rueda F, Cespedes MV, Conchillo-Sole O, Sanchez-Chardi A, Seras-Franzoso J, Cubarsi R, et al. Bottom-Up Instructive Quality Control in the Biofabrication of Smart Protein Materials. Advanced materials 2015;27:7816-22.

[4] Unzueta U, Ferrer-Miralles N, Cedano J, Zikung X, Pesarrodona M, Saccardo P, et al. Non-amyloidogenic peptide tags for the regulatable self-assembling of protein-only nanoparticles. Biomaterials 2012;33:8714-22.

Funding: We are indebted to Agencia Estatal de Investigación (AEI) and to Fondo Europeo de Desarrollo Regional (FEDER) (grant BIO2016-76063-R, AEI/FEDER, UE) to AV, AGAUR (2017SGR-229) to AV and 2017SGR-865 GRC to RM; CIBER-BBN (project NANOPROTHER) granted to AV and CIBER-BBN project 4NanoMets to RM; ISCIII (PI15/00272 co-founding FEDER) to EV and ISCIII (Co-founding FEDER) PIE15//00028 and PI18/00650 to RM, and to EU COST Action CA 17140. We are also indebted to the Networking Research Center on Bioengineering, Biomaterials and Nanomedicine (CIBER-BBN) that is an initiative funded by the VI National R&D&I Plan 2008–2011, Iniciativa Ingenio 2010, Consolider Program, CIBER Actions and financed by the Instituto de Salud Carlos III

## P12. Ceno-antibiogram: Biotechnological application of the antibiogram techniques for the analysis of the antibiotic-resistance of the gut-microbiota after fructose consumption

### Eduardo Gomez Casales^1^, Elena Fauste Alonso^2^; Marina Robas Mora^3^; Jesus presa^5^; Paola Otero Gómez^2^; María Isabel Panadero Antón^2^; Pedro Antonio Jiménez Gómez^3^; Agustín Probanza Lobo^4^; Carlos Bocos de Prada^2^

#### ^1^ Student. Faculty of Pharmacy, Universidad San Pablo CEU, Boadilla del Monte, Madrid, Spain; ^2^Biochemistry Area, Pharmaceutical and Health Sciences Department, Faculty of Pharmacy, Universidad San Pablo CEU, Boadilla del Monte, Madrid, Spain; ^3^Microbiology Area, Pharmaceutical and Health Sciences Department, Faculty of Pharmacy, Universidad San Pablo CEU, Boadilla del Monte, Madrid, Spain; ^4^Plant physiology Area, Pharmaceutical and Health Sciences Department, Faculty of Pharmacy, Universidad San Pablo CEU, Boadilla del Monte, Madrid, Spain; ^5^ Without ascription

##### **Correspondence:** Eduardo Gomez Casales (e.gomez53@usp.ceu.es)

Fructose consumption has increased considerably in the last decades associated with its incorporation in sugary drinks and processed foods [1]. This increase in consumption has been related to cardiovascular diseases, obesity and diabetes [2]. Recently, it has been shown that the gut microbiota is directly related to diet. This link affects both the functional activity and the composition of the gut microbiota [3]. Thus, the consumption of this sugar could affect the expression and profile of antibiotic resistance of said microbiota. This fact evidences the growing scientific interest in knowing those factors that may negatively affect the increase in antibiotic resistance, among which could potentially include the effect of this sweetener on the gut microbiota and its consequences.

The contribution of the present work consists of the biotechnological adaptation of techniques traditionally used for the evaluation of antibiograms of clinical isolates (phenotypic expression of population resistance) for the study of the behavior of the intestinal microbiota against antibiotics the coeno-antibiogram [4], insofar as it concerns the study of the phenotypic expression of the antibiotic resistance of the bacterial community as a whole (“coeno-“ from greek κοινός koinós 'common'). In this study, male Sprague-Dawley rats were used from mothers who received water during pregnancy (*CONTRO*L) or mothers who consumed fructose in the drinking water (*FRUCTOSA*) who, after reaching adulthood, consumed fructose for 21 days (CF and FF), fructose with salt (components of the Western diet) (CX and FX) or water (CC and FC) together with a standard solid diet. The coeno-antibiogram (Epsilon test© and Vitek©) was studied in the feces of these animals. For the treatment of the data the Random Forest and Wilcoxon statistics were used.

The results of the gut microbial communities show an increase of Minimum Inhibitory Concentration (MIC) according to the following model: (CF) against piperacillin and amoxicillin; (FF) against cefuroxime and imipenem and (FX) against amoxicillin. Increases that, however, are not observed in the other groups (CC and CX).

References

1. Tappy, Luc y Lê, Kim-Anne Lê. “Health effects of fructose and fructose-containing caloric sweeteners: where do we stand 10 years after the initial whistle blowings?.” *Current diabetes reports vol. 15*, 2015

2. Riveros, María Jesús, Parada, Alejandra y Pettinelli, Paulina. "Consumo De Fructosa y Sus Implicaciones Para La Salud: Malabsorción De Fructosa e Hígado Graso no Alcohólico." *Nutrición Hospitalaria 29.3*, 2014

3. Astbury, Stuart *et al.,* “High Fructose Intake During Pregnancy in Rats Influences the Maternal Microbiome and Gut Development in the Offspring.” *Frontiers in genetics vol. 9,* 2018

4. Robas Mora et al., “Effect of the Type of Vitis vinifera Cultivation in the Cenophenoresistome and Metabolic Profiling (CLPP) of Edaphic Bacterial Communities” *Journal of Agricultural Science and Technology A 7 (8),* 2017

Funding: Research support service (SAI-microbiología USPCEU)

## P13. Fur proteins as putative sensors of carbon/nitrogen balance in cyanobacteria: 2-oxoglutarate modulates the affinity of FurA for the *ntcA* promoter in *Anabaena sp.* PCC 7120

### Jorge Guío^1^, Cristina Sarasa^1^, Adrián Velázquez-Campoy^1,2,3^, M. Teresa Bes^1^, M. Luisa Peleato^1^, María F. Fillat^1^ and Emma Sevilla ^1^

#### ^1^ Department of Biochemistry and Molecular and Cell Biology and Institute for Biocomputation and Physics of Complex Systems, University of Zaragoza, Pedro Cerbuna 12, 50009 Zaragoza, Spain; ^2^ Aragon Institute for Health Research (IIS Aragon), Zaragoza, Spain; ^3^ CIBER of Hepatic and Digestive Diseases (CIBERehd), Madrid, Spain; ARAID Fundation, Government of Aragon, Zaragoza, Spain

##### **Correspondence:** Jorge Guío (jguiomartinez@gmail.com)

FurA (Ferric Uptake Regulator) from the cyanobacteria *Anabaena sp.* PCC 7120 is a transcriptional regulator that controls not only iron homeostasis but also other important cellular processes such as oxidative stress response, nitrogen metabolism or cell morphology [1]. Furthermore FurA has been proposed to act as a redox sensor protein, due to its disulfide reductase activity and its ability to interact with heme [2]. 2-oxoglutarate (2-OG), a metabolite produced in the Krebs cycle, acts as a signal of carbon/nitrogen balance and, in cyanobacteria, it modulates DNA-binding activity of the key regulator for nitrogen metabolism NtcA [3]. As previous studies showed that FurA was able to control the transcription of *ntcA* and *hetR* genes [4] we wondered if this transcriptional regulator could also be sensing carbon/nitrogen balance via 2-OG. Isothermal Titration Calorimetry (ITC) assays proved that FurA was able to bind to 2-OG and Electrophoretic Mobility Shift Assays (EMSA) showed that FurA binding activity to the *ntcA* gene promoter region was modulated by this metabolite. In order to predict its binding site, a model of FurA tridimensional structure was built and docked with 2-OG, revealing that the potential binding site of this molecule was close to DNA binding domain and contained basic aminoacids. Multiple sequence alignments showed that these residues were present in Fur proteins from other cyanobacteria species, such as *M. aeruginosa* or *N. spumigena*, and docking simulations predicted that 2-OG was also able to bind to these structures. Taken together, these results suggest a putative role of Fur proteins as sensors of carbon/nitrogen balance in cyanobacteria.

References

1. González A, Angarica V, Sancho J, Fillat M. The FurA regulon in *Anabaena sp.* PCC 7120: in silico prediction and experimental validation of novel target genes. Nucleic Acids Research. 2014;42(8):4833-4846

2. Fillat M. The FUR (ferric uptake regulator) superfamily: Diversity and versatility of key transcriptional regulators. Archives of Biochemistry and Biophysics. 2014;546:41-52

3. Laurent S, Chen H, Bedu S, Ziarelli F, Peng L, Zhang C. Nonmetabolizable analogue of 2-oxoglutarate elicits heterocyst differentiation under repressive conditions in *Anabaena sp.* PCC 7120. Proceedings of the National Academy of Sciences. 2005;102(28):9907-9912

4. González A, Valladares A, Peleato M, Fillat M. FurA influences heterocyst differentiation in *Anabaena sp.* PCC 7120. FEBS Letters. 2013;587(16):2682-2690

Funding: This work was supported by grants E35_17R from Gobierno de Aragón and BFU2016-77671-P/FEDER from MINECO

